# Fast Segmentation of Stained Nuclei in Terabyte-Scale, Time Resolved 3D Microscopy Image Stacks

**DOI:** 10.1371/journal.pone.0090036

**Published:** 2014-02-27

**Authors:** Johannes Stegmaier, Jens C. Otte, Andrei Kobitski, Andreas Bartschat, Ariel Garcia, G. Ulrich Nienhaus, Uwe Strähle, Ralf Mikut

**Affiliations:** 1 Institute for Applied Computer Science (IAI), Karlsruhe Institute of Technology, Karlsruhe, Germany; 2 Institute for Toxicology and Genetics (ITG), Karlsruhe Institute of Technology, Karlsruhe, Germany; 3 Steinbuch Center for Computing (SCC), Karlsruhe Institute of Technology, Karlsruhe, Germany; 4 Institute of Applied Physics (APH) and Center for Functional Nanostructures (CFN), Karlsruhe Institute of Technology, Karlsruhe, Germany; 5 Department of Physics, University of Illinois at Urbana-Champaign, Urbana, Illinois, United States of America; University of Campinas, Brazil

## Abstract

Automated analysis of multi-dimensional microscopy images has become an integral part of modern research in life science. Most available algorithms that provide sufficient segmentation quality, however, are infeasible for a large amount of data due to their high complexity. In this contribution we present a fast parallelized segmentation method that is especially suited for the extraction of stained nuclei from microscopy images, *e.g.*, of developing zebrafish embryos. The idea is to transform the input image based on gradient and normal directions in the proximity of detected seed points such that it can be handled by straightforward global thresholding like Otsu’s method. We evaluate the quality of the obtained segmentation results on a set of real and simulated benchmark images in 2D and 3D and show the algorithm’s superior performance compared to other state-of-the-art algorithms. We achieve an up to ten-fold decrease in processing times, allowing us to process large data sets while still providing reasonable segmentation results.

## Introduction

Recent developments of fluorescence microscopy techniques have revealed unprecedented possibilities for the *in vivo* analysis of developing specimens [1], [2]. Especially the lately established selective plane illumination microscopy (SPIM) and the even more advanced digital scanned laser light-sheet microscopy (DSLM) enable a detailed and comprehensive analysis of the early developmental stages of investigated model organisms such as the zebrafish (*Danio rerio*) or the fruit fly (*Drosophila melanogaster*) [3], [4]. The tremendous amount of acquired 3D+t spatio-temporal image data, however, cannot reasonably be analyzed manually. Therefore, highly automated procedures for the analysis of such biological image data have become an increasingly important component of current research in the life sciences [5]–[7]. For example in typical experiments, imaging the development of a zebrafish embryo within the first ten hours post fertilization (hpf) results in several thousand of 3D image stacks with file sizes of multiple Gigabytes per image stack [8], [9]. Thus, even a modest experiment with a single embryo easily accumulates multiple Terabytes of raw data.

Despite the development of tools for processing and storing large data sets, it still remains a challenge to accurately analyze large data sets in a reasonable amount of time. A frequently emerging task for this type of images is, for instance, the detection and segmentation of nuclei labeled with a fluorescent marker such as the green fluorescent protein (GFP) [10]. The properties of extracted nuclei can be used in a subsequent tracking step to associate corresponding objects in adjacent time frames. Such procedures provide insight into cellular ancestry and organogenesis of the evolving organism [11]. Current approaches to deal with the large amount of data are either a dramatic data reduction by specimen-dependent maximum intensity projections [12] or by using image compression and highly specialized GPU implementations [1]. In this contribution, we present a new segmentation algorithm that is specifically designed to perform a fast, parallelized extraction of stained nuclei from the raw 3D microscopy images on a usual desktop computer with the opportunity to execute it in a cluster environment. We confirm that our algorithm’s segmentation quality is comparable to other state-of-the-art nucleus segmentation methods, while also enabling large-scale data analysis that was impossible with currently available algorithms and implementations.

A typical analysis pipeline to extract fluorescently labeled nuclei in microscopy images is comprised of low-pass noise removal, such as Gaussian or median filtering, followed by a coarse object detection stage that identifies the objects of interest or the regions of interest in the images [13]–[17]. Detected objects, later referred to as seed points, can subsequently be used to perform a more thorough analysis of the image material in the segmentation step [16], [17]. Various algorithms for seed detection and segmentation have already been presented and were successfully applied for the detection of labeled nuclei. Seed detection methods range from relatively simple Euclidean distance map-based methods [18], over Laplacian-of-Gaussian (LoG) blob detection [13], [19] to shrinking level sets and other partial differential equation (PDE) based methods [20]. The segmentation step produces a binary mask that is used to extract meaningful features of the objects of interest and can be used for further quantitative analysis of the objects [21], [22]. Most straightforward segmentation approaches such as Otsu’s method or the watershed transform yield poor segmentation results due to the low contrast, relatively low signal-to-noise ratio and the densely packed objects of interest [23]. More sophisticated algorithms such as level set [24], graph based formulations such as graph cut [15], [17], [25] or gradient flow tracking [16], [26] methods provide good segmentation results but may become infeasible for high-throughput analyses due to their complexity and the accompanied high demands on computational resources.

For subsequent tracking steps that rely on the segmentation results it is often sufficient to have rough estimates of cell properties such as mean intensity, bounding volume and exact shape. Hence, instead of providing yet another high-quality segmentation algorithm, we focus on a trade-off between maximizing the quality of the obtained results while maintaining valuable performance of the calculations to enable high-throughput experiments. We use a LoG scale-space maximum intensity projection to identify seed points that correspond to the expected centroids of the nuclei [17]. This method was specifically chosen because it can be easily transferred to 3D blob detection and fast implementations based on recursive Gaussian filtering exist [27]. The fast approximate segmentation uses angular information between nucleus normals and the smoothed gradient at pixel locations in the proximity of the seed point. Additionally, the pixels are weighted according to their distance to the seed point by using Gaussian-based smoothing kernels. The efficiency of the algorithm helps to reduce the time needed for the analysis of Terabyte-scale experiments from several days to a few hours, *i.e.*, by a factor of up to ten compared to previous methods. Simultaneously, the segmentation quality is sufficient to perform further statistical analysis of the specimens. The remainder of this paper covers the methodology we use to rapidly identify seed points and describes the method and the fast parallel implementation of our segmentation algorithm. In the results section we demonstrate the quality of the introduced method on a suitable 2D benchmark data set from the Broad Bioimage Benchmark Collection and a realistic 3D data set based on simulated image material by Svoboda *et al.* [28]. To assess the computational efficiency, we compare the performance of our algorithm to several other well-established algorithms described in the methods section on differently scaled 3D image stacks of a zebrafish embryo and qualitatively compare the achieved 3D segmentation quality.

## Methods

### Seed Detection

Reliable and reproducible detection of seed points, such as the identification of approximate centroid positions of stained nuclei in microscopy images, is a mandatory component of most seed-based segmentation algorithms. The major benefit of the seed detection step is to be able to significantly constrain the regions of interest that are further investigated by the segmentation method and thus to minimize the memory and processing time consumption of the automated analysis. Moreover, detected seed points are used to guide the more complex segmentation methods to the region of interest. Similar to the work of Al-Kofahi *et al.* [17], we make use of the LoG blob detector with different scales to find spherical objects in the image. The LoG and its approximations Difference-of-Gaussian (DoG) or Difference-of-Mean (DoM) are well established edge and blob detection methods in the image analysis community and can be easily parameterized for the detection of spherical objects [13]. Scale-space-based interest point detectors rely on the assumption that points of interest, such as stained nuclei in this case, are present at multiple scales with an intensity maximum at a specific size dependent scale. As we deal with 3D image data, we use a scale-normalized 3D filtering approach that considers physical spacing of voxels:
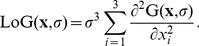
(1)with 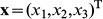 and 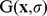 representing a Gaussian filtered image with standard deviation 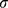. For performance reasons, however, we skip the Euclidean distance map-based automatic scale selection performed by Al-Kofahi *et al.* and restrict the calculations to a predefined set of scales. Using the relationship that the radius of detected objects 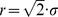, the appropriate set of scales can be determined by *a priori* knowledge about the investigated biological content of the images [17]. Here, we measured minimal and maximal radii of nuclei in pixels and directly used these values to attain 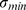 and 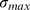 for the LoG filtering, respectively. Of course, it is important to consider the physical size of the pixels in the case of anisotropic image acquisition. The parameters used for the algorithmic validation are provided in the results section. A common approach to detect centroids of these objects is to search for intensity maxima in the spatial neighborhood of each pixel as well as in the neighboring scales as described in [13], [19]. Due to the enormous image size of several Gigabytes, however, it is not feasible to keep multiple image stacks of the scale-space simultaneously in memory. Therefore, we make use of an iteratively calculated LoG scale-space maximum intensity projection with a predefined discrete step size, *i.e.*, we calculate an image of the form:



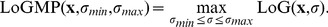
(2)The image generated according to Eq. 2 is an additional intensity image that stores the maximum intensity value attained for multiple filtering steps with different standard deviations 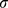 that were used for the LoG convolution operation. To preserve the information, which of the standard deviations used for LoG filtering was responsible for the maximum value in the image attained by Eq. 2, we additionally store the scale that yielded the maximum value at each pixel location:
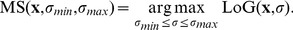
(3)


**Figure 1:**
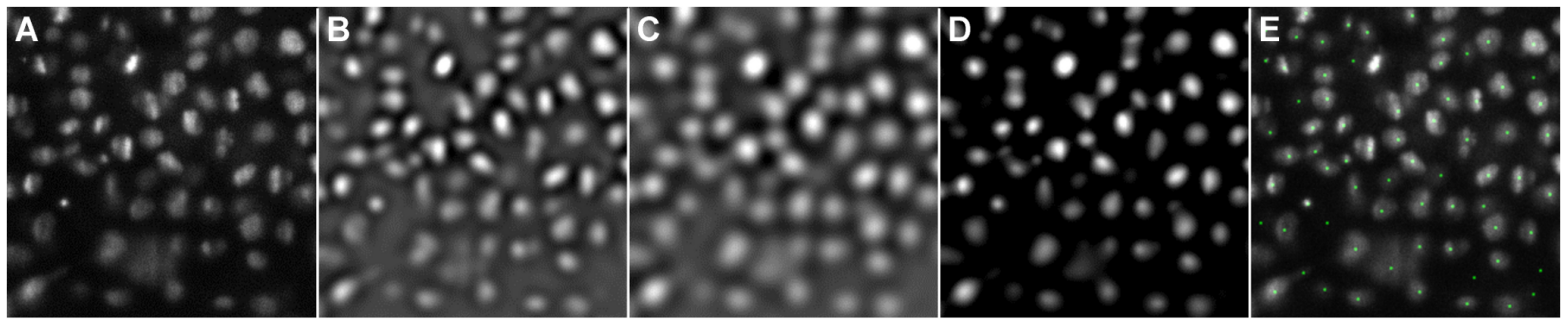
Processing steps for the generation of a LoG scale-space maximum intensity projection used for 2D seed detection. Original image (A), LoG filtered image with 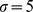 and 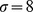 (B, C), LoG scale-space maximum intensity projection with 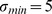, 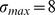 and 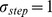 (D) and the detected seeds plotted on the original image (E).

The information stored in the maximum scale image described by Eq. 3 is beneficial for further processing steps as it directly provides an initial size estimate of the object about to be extracted. Finally, the actual seed extraction from the LoG scale-space maximum intensity projection image comes down to a simple local extrema detection in the direct neighborhood of each pixel (8-neighborhood and 26-neighborhood for 2D and 3D images, respectively). Close maxima that are likely to belong to a single nucleus are fused, and a noise reduction is performed by applying an intensity threshold to discard dark seed points on the background. Fig. 1 exemplarily depicts the processing steps needed to attain the LoG scale-space maximum intensity projection for a 2D image and highlights the detected seed points.

### Fast Approximate Segmentation of Roundish Objects

The key idea of the proposed fast segmentation method is to transform the input image or rather regions surrounding the detected seed points to a representation that can be handled by straightforward algorithms like Otsu’s method [23]. The first step of the segmentation algorithm is to homogeneously distribute the seed points among different threads and independently perform further calculations in parallel. A region around each seed point is cropped from the original image, in order to process as few pixels as possible. The size of this region is determined by the initial size estimate provided by the preceding seed detection step. Currently, we use a cuboid with side lengths of 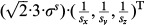, where 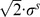 is the radius of the respective seed point at scale 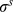 and 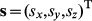 corresponds to the physical spacing of the voxels. For each pixel of the cropped region, the Gaussian smoothed gradient is calculated, *i.e.*,
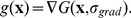
(4)


For the image material considered in this work, a value of 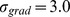 yielded satisfactory results. We calculate the difference vector of pixel 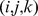 in the sub-region to the respective seed position 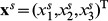 as
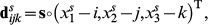
(5)with 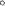 being the Hadamard product, and define the normal 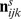 at each pixel location as



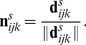
(6)The normal corresponds to a vector pointing from the seed point location to the considered pixel. The next step is to calculate the dot product of each normal in the cropped region with the corresponding normalized gradient vector
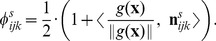
(7)


**Figure 2:**
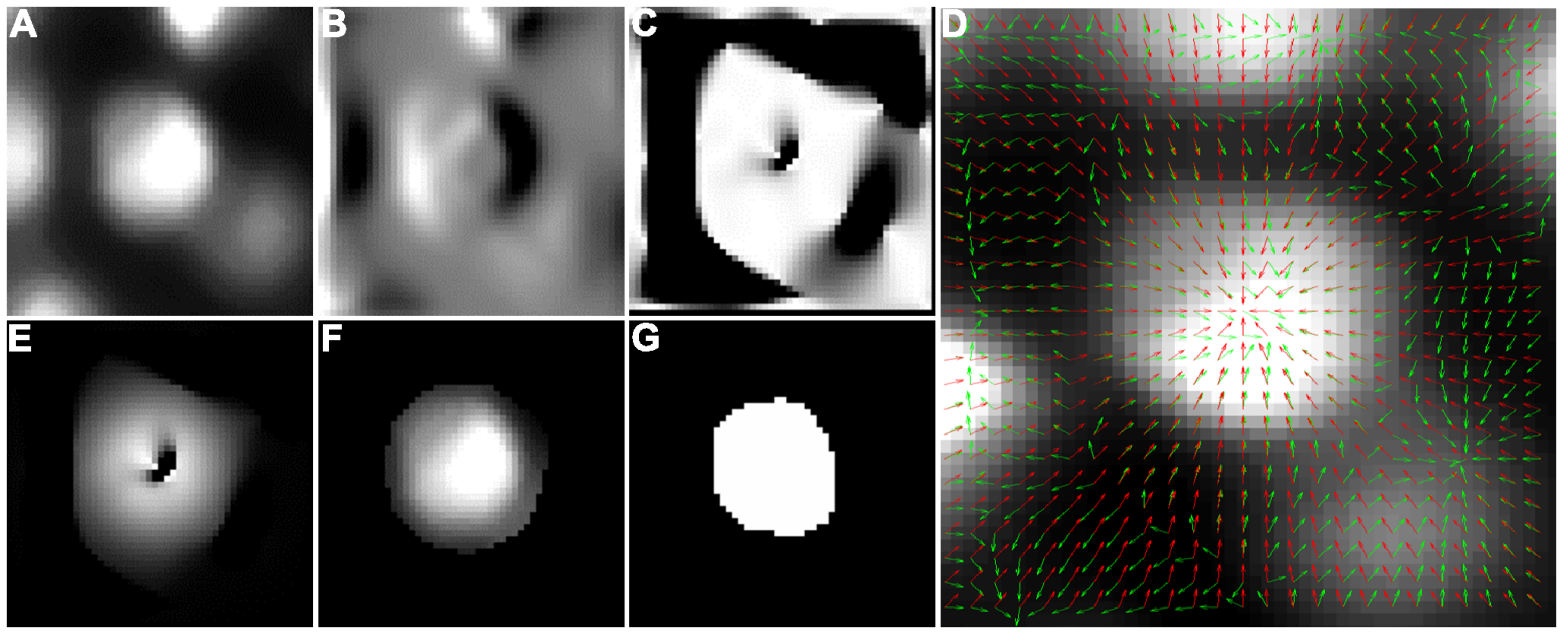
Processing steps that are performed in parallel for each detected seed point. Cropped raw image (A), Gaussian smoothed left-right derivative image (B), dot product of the normalized gradient with the seed normal (C), raw image with smoothed gradient and normal vector field overlay (D), weighted version of the previously calculated dot product (E), resulting intensity image (F) and the final segmentation result (G).

This contrast invariant measure is similar to the one described by Soubies *et al.* [29], where it is used in the energy term of an ellipsoid fit segmentation approach. The transformed dot product of normalized vectors in Eq. 7 yields only values in the interval 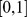, with 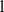 being identical, 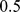 being perpendicular and 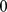 being opposing vectors. As depicted in panels Fig. 2C,D, this property can be exploited to discard pixels in the vicinity of the seed point that clearly belong to neighboring cells.

Additionally, we decrease the intensity value of pixels in the sub-region that are far away from the detected seed location. Based on the initial radius estimation, *i.e.*, 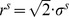 and a Gaussian kernel standard deviation 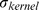, we define the following weighting function:
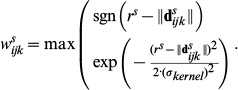
(8)


**Figure 3:**
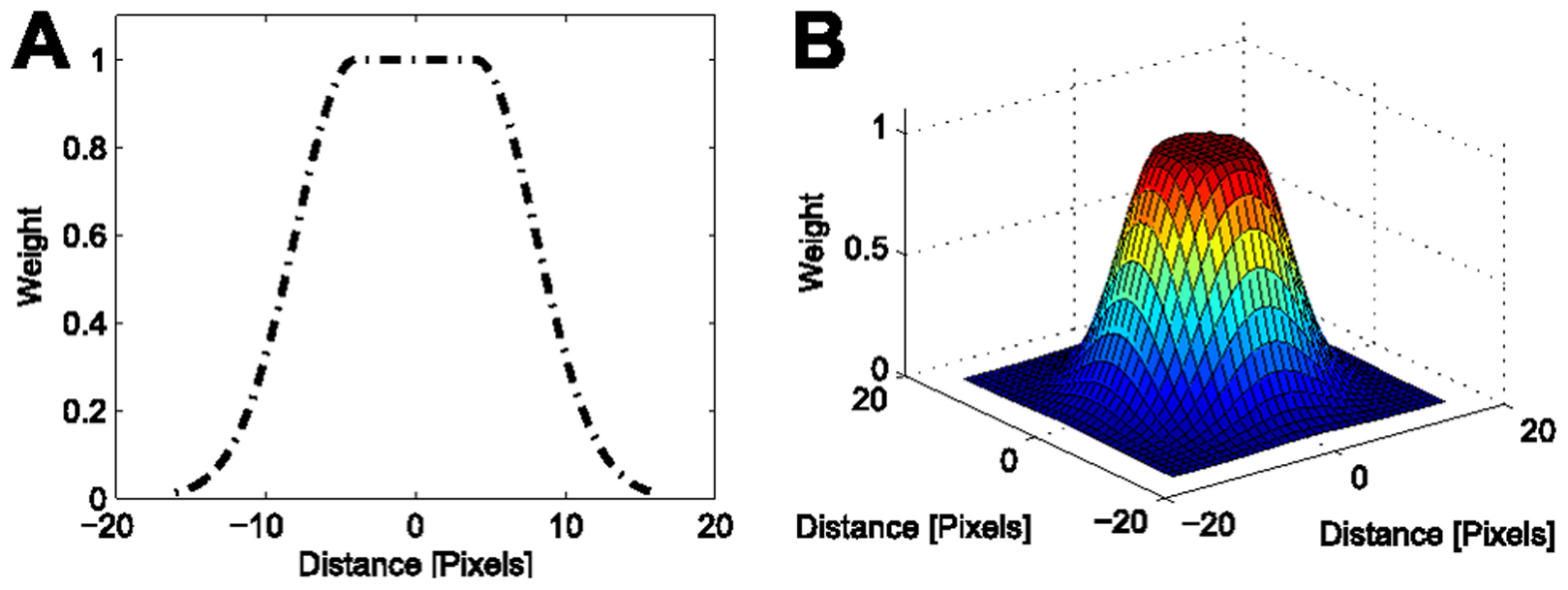
Exemplary weighting kernel for 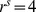 depicted in 1D (A) and 2D (B). The kernel should be chosen such that the region of interest yields high weights.

The weighted normalized dot product, shown in panel Fig. 2E, is calculated according to the pixel-wise multiplication 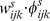. To combine the cropped raw image (Fig. 2A) and the weighted dot product image (Fig. 2E), the original intensity values are copied within the seed radius and all remaining raw intensity values are multiplied with the weighted dot product image. The result of this operation is shown in Fig. 2F. Applying Otsu’s method on the weighted and cropped original image depicted in Fig. 2F yields the final segmentation shown in Fig. 2G. In the present implementation we used a Gaussian-based smoothing kernel as depicted in Fig. 3. The plateau in the centre of the kernel is determined by the initial size estimation of the seed detection stage and corresponds to regions that are likely to belong to the nucleus of interest. To be able to adjust the algorithm for different segmentation scenarios, the weighting kernel plateau region radius 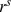 can optionally be scaled using a multiplier, which is set to 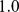 by default. Similarly, the degree of flattening of the kernel for larger distances to the centre can be controlled using the kernel standard deviation 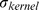.

As the properties of segmented regions are immediately extracted from the cropped image, a final labeling step becomes redundant. Another benefit of this direct information extraction is that literally no merged nuclei appear, which is a frequent problem of other segmentation algorithms. A subsequent watershed transform to separate merged nuclei can therefore also be omitted. In the remainder of this paper we refer to our new algorithm as TWANG (**T**hreshold of **W**eighted intensity **A**nd seed-**N**ormal **G**radient dot product image).

### Implementation Details

In the following section, we compare our algorithm (TWANG) to Otsu’s method (OTSU) [23], Otsu’s method with a watershed-based splitting of merged blobs (OTSUWW) [23], [30], a geodesic active contours method (GAC) [31], a gradient flow tracking segmentation (GFT) [16], [26] and a graph cut-based segmentation (GC) [17]. We selected Otsu’s method to demonstrate that the image material could not sufficiently be analyzed using straightforward adaptive thresholding. The remaining algorithms represent a variety of reasonable approaches for the segmentation of fluorescently labeled nuclei, as described in the respective publications [16], [17], [26], [30]. For OTSU and OTSUWW we used an additional Gaussian low-pass filter to reduce high frequency noise. The level set function of the GAC pipeline, was initialized using the LoG-based seed detection method described earlier.

The algorithms OTSU, OTSUWW, GAC and TWANG were implemented in a custom built C++ application using the Insight Toolkit SDK (http://www.itk.org/) [32]. We thoroughly ensured that all involved image processing operators were optimally exploiting modern hardware architecture, *i.e.*, making use of parallel implementations where possible and reducing the memory footprint of the large image data sets to a minimum. Besides our own ITK-based implementations, we used the C++ implementation provided by Liu *et al.* (http://www.cbi-tmhs.org/ZFIQ/download.htm) and Li *et al.* (http://www.biomedcentral.com/1471-2121/8/40) for gradient flow tracking segmentation in 2D and 3D, respectively [16], [26]. For the graph cut segmentation we used the implementation shipped with the FARSIGHT Toolkit (http://www.farsight-toolkit.org/) [17]. The parameters for all algorithms were manually optimized to fit the different image sets and are summarized in Table 1. The entire source code of the proposed TWANG segmentation, installation instructions and an example data set are publicly available for download and can be obtained from the online supplementary material at the journal’s website.

**Table 1:** Parameterization of the Algorithms.

Algorithm	Parameter	2D Benchmark	3D Benchmark	3D DSLM Images
OTSU [23]	Gaussian Std. Dev.	1.0	1.0	1.0
OTSUWW [23], [30]	Gaussian Std. Dev.	1.0	1.0	1.0
	Watershed Level	1.0	1.0	1.0
GFT [16]	Fusion Threshold	3.0	3.0	3.0
	Minimum Region	100	3000	50
	Diffusion Iterations	30	10	15
	Sigma	3.0	0.0	1.0
GAC [31]	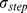 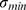 , 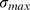 ,	8,11,1	10,13,1	6,9,3
	Propagation Scaling	0.8	0.8	0.5
	Curvature Scaling	0.55	0.55	0.05
	Advection Scaling	5.0	8.0	1.0
	Iterations	250	100	110
TWANG	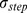 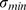 , 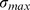 ,	8,11,1	10,13,1	6,9,3
	Gradient Image Std. Dev.	3.0	3.0	3.0
	Kernel Size Multiplier	1.5	1.2	1.5
	Kernel Std. Dev.	3.0	1.0	3.0

The parameter sets that were used to perform the segmentation comparisons of OTSU, OTSUWW, GFT, GAC, GC and TWANG. For a detailed description of the respective parameters refer to the original papers of the algorithms. The graph cut segmentation (GC) implemented in the FARSIGHT Toolkit worked out of the box with automatic parameter selection and therefore used individual parameters for each image.

### Evaluation Criteria and Benchmark Images

To verify the quality of achieved segmentation results we compared our new algorithm to multiple well established segmentation algorithms. The evaluation was based on the criteria described by Coelho *et al.* in [33], namely the Rand Index (RI), the Jaccard Index (JI), the Normalized Sum of Distances (NSD) and the Hausdorff Metric (HM). The RI is defined as the fraction of index pairs that have the same labeling in reference and segmentation versus all possible pixel pairs and is given as a percentage (100% for perfect agreement). The JI is similar to the RI and is determined by the fraction of matching pixel pairs versus all cases of non-matching pairs. JI is not upper-bound (higher values are better). The HM is defined as the maximum of the set of minimal distances of two compared shapes (lower values are better) [34]. The NSD reflects the average distance of labeled pixels that do not agree in reference and segmentation (lower values are better) [33]. Furthermore, the number of added, missing, erroneously split and merged segments are compared. For a detailed description of the criteria refer to [33].

We used a selection of thirty representative images of the image set BBBC006v1 from the Broad Bioimage Benchmark Collection (http://www.broadinstitute.org/bbbc) for 2D segmentation evaluation. The data set is similar to our target application of quantifying images of developing specimens and provides a complete set of labeled segmentation images that serve as a reliable ground truth. Moreover, we used a set of thirty 3D benchmark images containing simulated nuclei of a HL60 cell line with a low signal-to-noise ratio and a clustering probability of 75%, which were generated using the CytoPacq simulation toolbox by Svoboda *et al.* (http://cbia.fi.muni.cz/images/stories/user_images/david/datasets/HL60_HighNoise_C75_3D_TIFF.zip) [35]. The simulated image data was accompanied with a labeled ground truth and was therefore perfectly suited to perform the segmentation evaluation in 3D. Unfortunately, up to date no reliably annotated 3D microscopy images of labeled nuclei exist. Due to a missing gold standard algorithm and the enormous effort needed for manual segmentation of 3D images, we can only present qualitative segmentation results for this use case.

All calculations were performed on a desktop PC equipped with an Intel Core i7–2600 CPU @ 3.4 GHz and 32 GB of memory installed. Processing times were measured in seconds.

## Results

**Figure 4:**
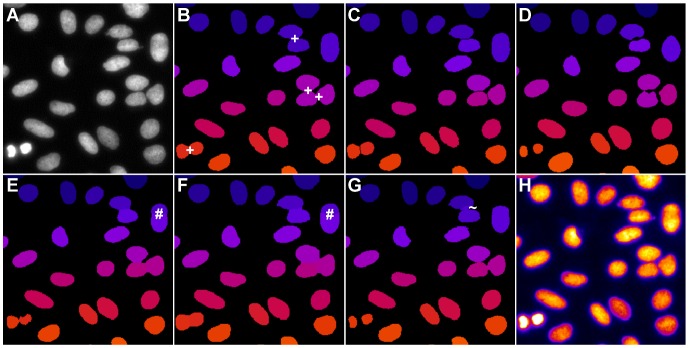
Comparison of the segmentation quality achieved by the investigated algorithms on 2D benchmark images from the Broad Bioimage Benchmark Collection (BBBC006v1). Original image (A), adaptive thresholding using Otsu’s method [23] (B), Otsu’s method combined with watershed-based blob splitting [23], [30] (C), geodesic active contours [31] (D), gradient vector flow tracking [16] (E), graph-cuts segmentation [17] (F), TWANG segmentation (G) and a false colored original image (H). The symbols indicate segmentation errors for nuclei that are either split (#), merged (+), missing (o) or spurious (∼).

The results of the quality comparison for the 2D benchmark are listed in Table 2 and Fig. 4. OTSU as well as the GAC exhibited many merged regions and may require post-processing steps to split merged objects. One such post-processing method using a watershed-based splitting of merged blobs was implemented in the OTSUWW pipeline, which showed a significantly reduced number of merged nuclei. GFT and GC worked properly for this set of images. Our proposed method seemed to be positioned on a good average position in the quality comparison and yielded the best values for HM and NSD. Additionally, the fact that very few merged regions were present in the segmentation results of the TWANG segmentation make it well suited for subsequent tracking tasks. An exemplary segmentation outcome of the investigated algorithms is depicted in Fig. 4. Except OTSU, all segmentation results were adequate. Problems that occurred even for such relatively easy images, were merged regions (Fig. 4B), split nuclei (Fig. 4E, F) and too large image regions (Fig. 4G). For the cropped region depicted in Fig. 4, OTSUWW (Fig. 4C) offered the best segmentation quality. Although the fastest algorithm in this case was OTSU, it could not reasonably be applied without the post-processing step. OTSUWW and TWANG segmentation offered the best trade-off between speed and quality.

**Table 2:** Comparison of the 2D segmentation quality.

Algorithm	RI	JI	HM	NSD(×10)	Split	Merged	Added	Missing	t [s]	t [s]*
OTSU	92.60	2.48	7.97	2.11	0.57	10.00	6.30	0.47	**0.10**	**0.09**
OTSUWW	92.54	2.48	6.20	1.70	3.13	1.57	6.67	0.53	0.25	0.25
GFT	92.58	2.48	6.70	1.54	4.03	**0.03**	4.27	1.70	0.45	–
GAC	90.41	2.36	6.60	1.43	**0.10**	5.13	**0.20**	16.03	1.64	0.54
GC	**96.71**	**2.58**	6.18	1.54	8.70	0.10	5.17	**0.03**	0.42	–
TWANG	92.27	2.48	**6.15**	**1.32**	1.10	1.03	3.30	7.17	0.35	0.15

Comparison of the segmentation quality on 2D benchmark images from the Broad Bioimage Benchmark Collection (BBBC006v1). For quality assessment we used the Rand Index (RI), the Jaccard Index (JI), the Hausdorff Metric (HM) and the Normalized Sum of Distances (NSD) as defined in [33]. Besides correct segmentations, nuclei can be split, merged, erroneously added or are missing. The listed values are the arithmetic mean values of 30 processed 2D benchmark images. Performance of the algorithms was tested without using threads and with 8 threads where possible (indicated by *).

**Figure 5:**
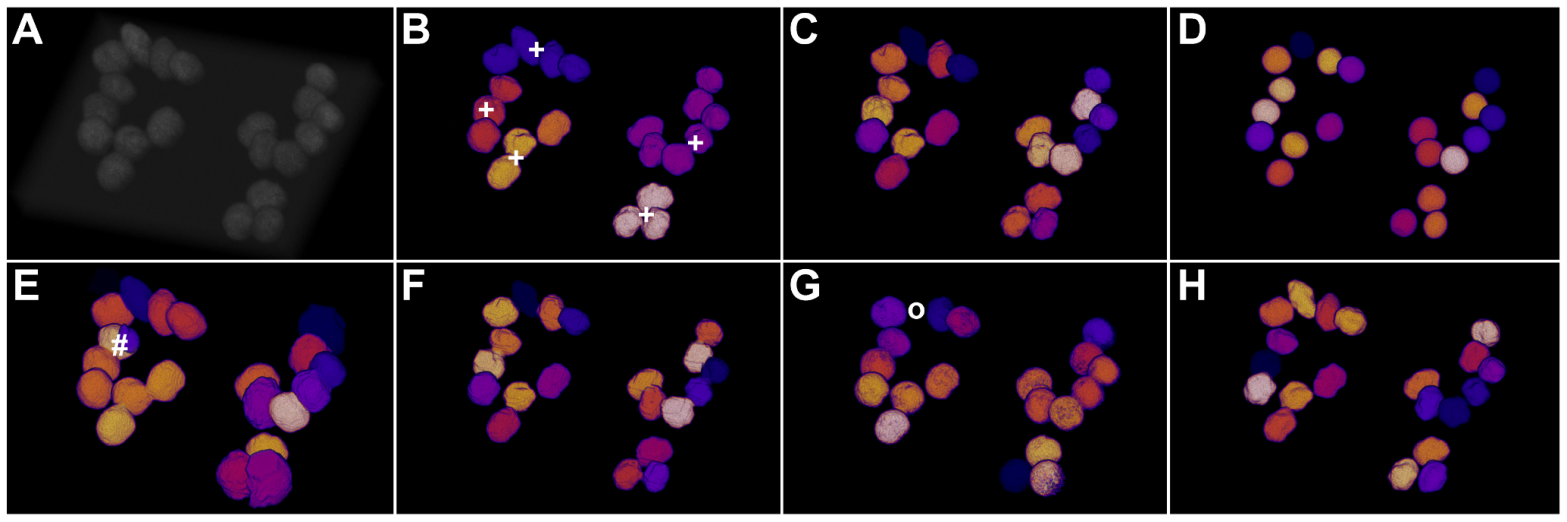
Comparison of the segmentation quality achieved by the investigated algorithms on simulated 3D benchmark images by Svoboda *et al.* (HL60 cell line, low SNR, 75% clustering probability)[35]. Simulated original image (A), adaptive thresholding using Otsu’s method [23] (B), Otsu’s method combined with watershed-based blob splitting [23], [30] (C), geodesic active contours [31] (D), gradient vector flow tracking [16] (E), graph-cuts segmentation [17] (F), TWANG segmentation (G) and the simulated ground truth image (H). The symbols indicate segmentation errors for nuclei that are either split (#), merged (+), missing (o) or spurious (∼).

In Table 3, the quality comparison on the simulated 3D benchmark data is listed. For this data set, the optimal values are distributed quite heterogeneously and most algorithms produce acceptable results. OTSU performed poorly again and yielded many merged regions (Fig. 5B), which could be perfectly corrected by the watershed-based post-processing (Fig. 5C). GAC failed to nicely extract the shape of the nuclei due to poor edge information and GFT tended to produce segments that were too large. Different parameterizations resulted in heavy under- or over-segmentation in both cases. The graph cut implementation provided good segmentation results and although regions may not be captured as accurately as with the watershed-corrected adaptive thresholding, the splitting of single nuclei was performed properly in most cases. The time needed to process the images significantly varied between the algorithms. GFT, for example, was up to one order of magnitude slower than OTSU, OTSUWW and TWANG. At the same time, GFT did not deliver a more convincing segmentation quality that would justify the slower execution. Fig. 5 shows 3D volume renderings of the false-colored segmentation results. Highlighted errors correspond to merged regions (Fig. 5B), split regions (Fig. 5E) and missing objects (Fig. 5G). TWANG and OTSUWW again seemed to offer the best compromise of speed vs. quality.

**Table 3:** Comparison of the 3D segmentation quality.

Algorithm	RI	JI	HM	NSD(x10)	Split	Merged	Added	Missing	t [s]	t [s]*
OTSU	97.35	6.00	22.82	5.72	**0.00**	3.27	0.87	**0.00**	**0.49**	**0.44**
OTSUWW	97.57	6.03	**3.80**	**1.12**	0.13	**0.00**	**0.00**	**0.00**	2.57	2.48
GFT	88.06	3.57	6.81	6.25	0.10	1.57	6.53	1.87	15.51	–
GAC	95.06	**6.40**	7.41	2.52	**0.00**	1.13	**0.00**	0.77	5.92	–
GC	**97.78**	6.37	5.66	1.69	1.34	0.07	**0.00**	**0.00**	5.92	–
TWANG	93.82	4.94	6.62	2.41	**0.00**	**0.00**	**0.00**	1.37	3.72	1.08

Comparison of the segmentation quality on simulated 3D benchmark images by Svoboda *et al.* (HL60 cell line, low SNR, 75% clustering probability) [35]. For quality assessment we used the Rand Index (RI), the Jaccard Index (JI), the Hausdorff Metric (HM) and the Normalized Sum of Distances (NSD) as defined in [33]. Besides correct segmentations, nuclei can be split, merged, erroneously added or are missing. The listed values are the arithmetic mean values of 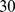 processed 3D benchmark images. Performance of the algorithms was tested without using threads and with 8 threads where possible (indicated by *).

**Figure 6:**
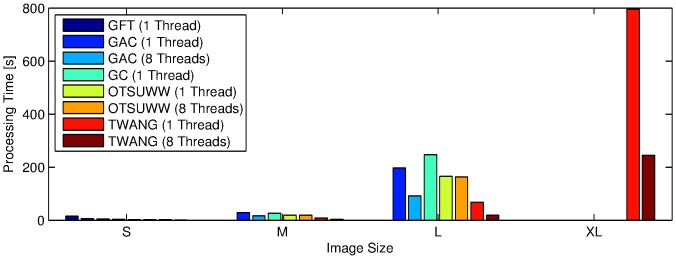
Bar plot of the measured processing times in seconds (lower values are better). Image sizes correspond to 256×256×50 (S), 512×512×100 (M), 1024×1024×200 (L) and 2048×2048×400 (XL) voxels. Missing bars indicate that the respective algorithms failed to process the given image size. TWANG segmentation turned out to be the fastest algorithm in all tested categories and was the only method that was able to process the XL images with the given memory constraint of 32 GB.

**Figure 7:**
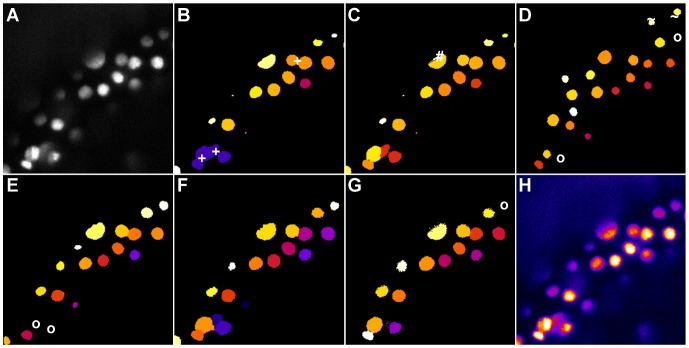
Comparison of the segmentation quality achieved by the investigated algorithms on a 3D image of labeled nuclei of a zebrafish embryo acquired using DSLM. The panels show the maximum intensity projection of 3 neighbouring z-slices. Original image (A), adaptive thresholding using Otsu’s method [23] (B), Otsu’s method combined with watershed-based blob splitting [23], [30] (C), geodesic active contours [31] (D), gradient vector flow tracking [16] (E), graph-cuts segmentation [17] (F), TWANG segmentation (G) and a false colored original image (H). The symbols indicate segmentation errors for nuclei that are either split (#), merged (+), missing (o) or spurious (∼).

The quantitative comparison of the achieved segmentation quality that was presented in the previous paragraphs confirmed the comparable segmentation quality achieved by our algorithm. All following evaluations were performed on our main target image material, *i.e.*, 3D image stacks of a zebrafish embryo that were acquired using DSLM. As a major motivation for our algorithm was to provide a significant reduction of processing times, we next investigated the computational efficiency of the different algorithms. Owing to the fact that OTSU proved to perform poorly without the watershed-based post-processing step, we omitted it for further performance tests. Table 4 and Fig. 6 summarize the measured processing times that were required to segment differently sized 3D image stacks by OTSUWW, GAC, GFT, GC and TWANG. The image stacks were cropped from a single time point of a zebrafish data set and had resolutions of 256×256×50, 512×512×100, 1024×1024×200, 2048×2048×400 voxels for sizes S, M, L and XL, respectively. Processing times represent the time needed to process a single stack of the respective image sizes. The proposed method clearly dominated the other algorithms for all investigated image sizes and was up to ten times faster. Even using a non-threaded implementation, the performance benefit of TWANG held true. Although the 2D and 3D benchmark images suggested to prefer OTSUWW, it was 3-fold slower than TWANG for larger 3D images. Furthermore, our method benefited heavily from threaded processing and achieved an up to 10-fold decrease of processing time, compared to OTSUWW. The XL image category could only be processed by TWANG, as the other algorithms exceeded the memory limitation of 32 GB. Concurrently, the quality of obtained segmentations was comparable to the results of the more complex algorithms. In Fig. 7, a maximum intensity projection of three neighbouring z-slices are shown for the segmentation results of each algorithm, which confirms our approach is comparable to other segmentation routines. In addition to the fast parallelized processing of small image regions, the memory footprint was kept small and did not increase with the image size. Due to a lack of 3D ground truth, however, the segmentation quality could only be subjectively rated on the basis of selected slices, such as the ones depicted in Fig. 7.

**Table 4:** Comparison of 3D Processing Times in Seconds and Segmentation Quality.

Algorithm	S	S*	M	M*	L	L*	XL	XL*	Quality
GFT [16]	14.80	–	–	–	–	–	–	–	0
GAC [31]	5.16	4.07	28.43	16.41	197.23	91.15	–	–	–
GC [17]	3.31	–	26.35	–	246.26	–	–	–	++
OTSUWW [23], [30]	2.40	2.37	19.19	19.08	164.92	162.75	–	–	++
TWANG	**1.31**	**0.44**	**8.22**	**2.48**	**66.98**	**18.22**	**795.25**	**243.8**	+

Processing time in seconds and subjective quality measure of five algorithms implemented in C/C++ with respect to the image size. Missing values indicate either memory consumption of more than 32 GB (OTSUWW, GAC, GC) or incapabilities of the software (GFT). Tiled processing was disabled for our implementations to ensure comparable memory limitations. Performance of the algorithms was tested without using threads and with 8 threads where possible (indicated by *). Due to a lack of 3D ground truth for the DSLM images, we provide a qualitative evaluation using five categories 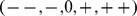.

**Figure 8:**
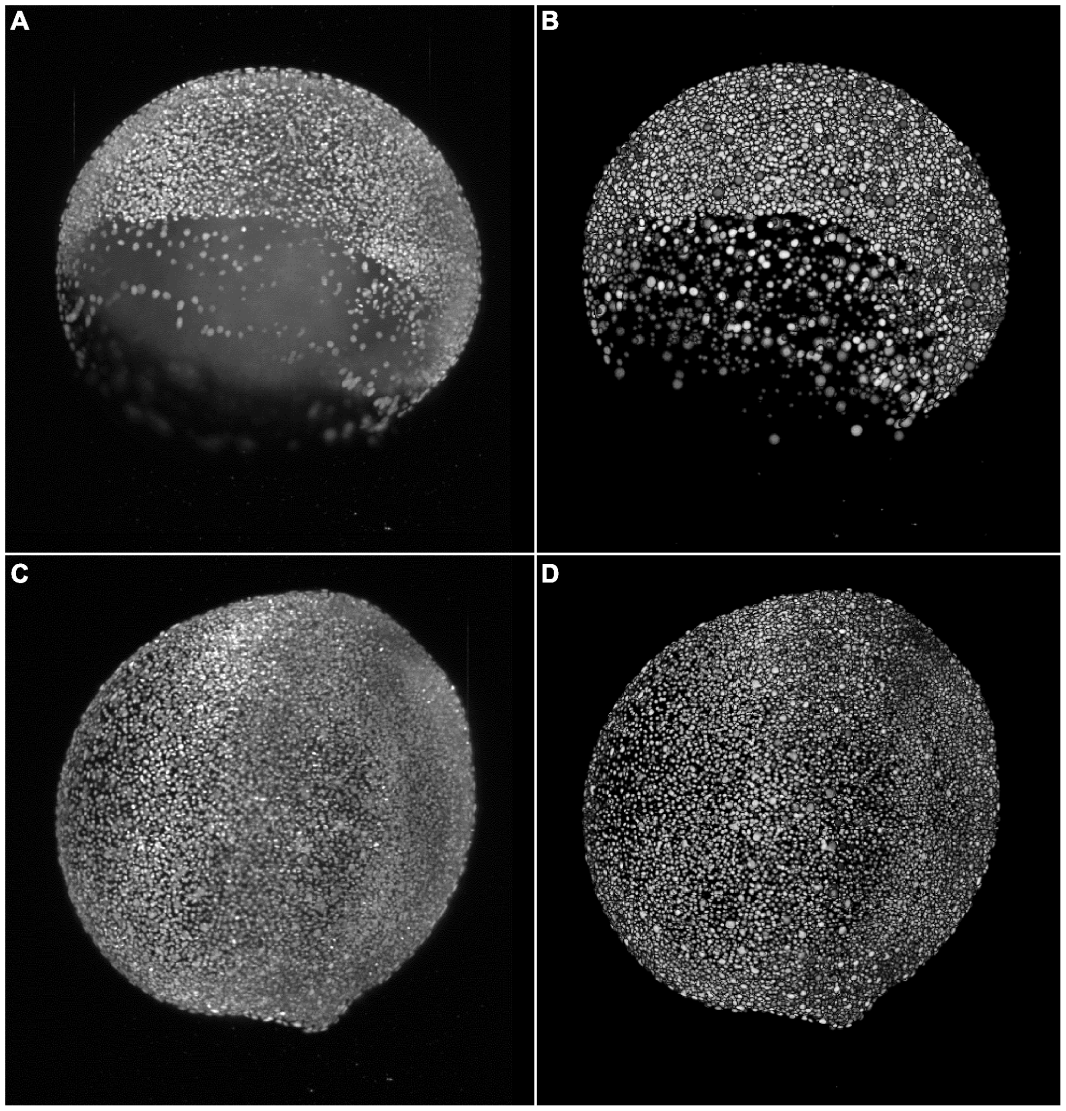
Results of the TWANG segmentation pipeline applied on two images of a developing zebrafish embryo. The images were captured at the 7
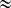
8000 cells (A,B) and at the 11 hpf stage with 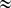
12000 cells (C,D), respectively. The panels show maximum intensity projections of the raw images (A,C) and the resulting segmentation using our TWANG segmentation pipeline (B,D). Each 3D image stack has a file size of 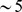
GB and comprises 2560×2160×500 voxels with a dynamic range of 16 bits. Processing one image stack takes approximately 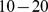 minutes on a common desktop machine, depending on the developmental stage of the embryo. Typical experiments may be comprised of up to 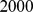 z-stacks (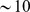
TB) for the spatio-temporal analysis of a single embryo.

An exemplary segmentation result for two time points of a developing zebrafish embryo that illustrate our target application are depicted in Fig. 8. The properties of the investigated specimen are well described and may serve as a basis for further processing steps such as tracking and cell lineage reconstruction. None of the other discussed algorithms was able to process images of such a size with the given implementation and memory constraints.

## Discussion

In this contribution we present a new segmentation method that is suitable for rapid extraction of information from large volumetric image data. It was shown that the proposed algorithm performed up to ten times faster than other established methods while still providing sufficient segmentation quality for subsequent analysis steps. We compared five well known methods for nucleus segmentation to our new algorithm both quantitatively and qualitatively on a 2D and a 3D benchmark data set. Additionally, we applied all algorithms on our target image material and showed that TWANG was the only method capable of providing a good trade-off between segmentation quality and fast performance.

Otsu’s method turned out to be the fastest of the considered algorithms. Due to it’s high tendency to produce merged segments, however, it was of no practical use without post-processing. Using a watershed-based splitting of the blobs attained by OTSU as performed with the OTSUWW implementation, the segmentation quality could be significantly raised. Regarding the segmentation quality, OTSUWW and GC provided the most convincing results. OTSUWW will presumably fail as soon as the blurring of the image material increases (e.g. in the axial direction of a 3D volume) or when nuclei are more clustered. Owing to the fact that OTSUWW, GFT, GAC and GC have a very high memory consumption and very limited possibilities for parallelization, none were appropriate for large 3D image stacks. While the memory limitation could be defeated using tiled processing of the images, this would dramatically decrease the performance of the algorithms due to an increased number of required read and write operations to the hard disk drive.

A general problem of seeded segmentation algorithms such as TWANG and GAC, though, is that the quality and reliability of extracted seeds directly influences the outcome of the segmentation. Especially, in regions close to the image border, our seed detection method missed some nuclei due to filtering artifacts that occurred if the convolution mask of a box filter did not completely fit into the processed image region. This behaviour was responsible for the increased number of missing nuclei observed for GAC and TWANG in the 2D segmentation benchmark. In most real applications this could simply be compensated by imaging the probe with sufficient padding in the border regions. In live specimens, observed nuclei are frequently dividing and therefore undergo changes in shape. As indicated, for instance, in Fig. 4G, our algorithm successfully extracts two separated smaller nuclei in the case of these events. The scale-space approach used for the seed detection is therefore well suited to identify seed points at different sizes and to provide this information to the segmentation.

For our target image data that was captured using DSLM microscopy, we recognized an increased amount of false positive seed detections upon reduced image quality caused by light scattering and absorption in image regions farther from the detection objective [36]. This problem mainly has to be solved at the acquisition stage, by using optimizations such as double-sided illumination, specimen rotation or optimally a more sophisticated multi-view acquisition [1], [37]. In addition, the extracted properties of segmented regions can be used to refine the results and to discard false positive detections based on criteria such as the integrated region intensity, seed intensity, volume, foreground vs. background ratio and the like. If subsequent tracking of detected nuclei is performed, the absence of a good matching partner in multiple neighbouring frames provides an additional indicator of having a false positive detection.

As confirmed by the quantitative and qualitative assessment of the segmentation quality, our algorithm produces comparable labeled images to other established methods. One problem that remains to be solved is the segmentation of strongly elongated structures. Due to the nature of the spherical weighting kernel, the algorithm tends to clip the tips of strongly elongated nuclei. In the current implementation, this can be compensated by adjusting the kernel size multiplier and the kernel standard deviation properly. In upcoming versions of the algorithm, this might be solved by estimating the elongation properties of nuclei directly at the seed detection stage in order to adapt the weighting kernels accordingly. The segmentation quality sacrificed by the high-performance implementation as well as the false positive rate for the seed detection stage may also be compensated by uncertainty propagation between all involved pipeline steps as described in [38].

All in all, the provided method represents a reasonable choice for a fast initial analysis of the data or for applications where it becomes infeasible to use methods such as [17]. Of course, if result accuracy of the extracted information is the main intention of an experiment, it might become inevitable to use more complex segmentation routines.

We aim to use the developed processing pipeline in upcoming projects to automatically analyze multiple Terabytes of experimental data of live zebrafish embryos that are stored on the Large Scale Data Facility (LSDF), a large distributed storage system offering multiple Petabytes of storage [39]. A distributed computing environment based on the Apache Hadoop framework is used to accelerate the automated analysis of the image data. A main goal will be to identify and quantify variations among different chemical treatments with respect to nucleus counts, nucleus densities, cell migration patterns, body part formation and other phenotypic alterations in space and time.

## Supporting Information

File S1 **Implementation of the TWANG Segmentation Algorithm.** C++ source code of the fast segmentation pipeline presented in this paper. The provided archive contains all sources, installation instructions and an example image.
(ZIP)

File S2 **Example Data of a Zebrafish Embryo.** The archive contains two additional cropped regions of a 3D DSLM image of a developing zebrafish embryo.
(ZIP)
