# Supplementary material for: Fast Segmentation of Stained Nuclei in Terabyte-Scale, Time Resolved 3D Microscopy Image Stacks
Source: PLoS One. 2014 Feb 27;9(2):e90036. doi: 10.1371/journal.pone.0090036 (PMC3937404; doi:10.1371/journal.pone.0090036)
Supplement: File S1 — Implementation of the TWANG Segmentation Algorithm. C++ source code of the fast segmentation pipeline presented in this paper. The provided archive contains all sources, installation instructions and an example image. (ZIP) [file pone.0090036.s001.zip › Software_S1/documentation/InstallationInstructions.pdf]

Fast Segmentation of Stained Nuclei in Terabyte-Scale 3D+T Microscopy Images - Installation Instructions

J. Stegmaier[1,*], J. C. Otte[2], A. Kobitski[2,4], A. Bartschat[1], A. Garcia[3], G. U. Nienhaus[2,4,5], U. Strähle[2], R. Mikut[1]
1 Institute for Applied Computer Science (IAI), Karlsruhe Institute of Technology, Karlsruhe, Germany
2 Institute for Toxicology and Genetics (ITG), Karlsruhe Institute of Technology, Karlsruhe, Germany
3 Steinbuch Center for Computing (SCC), Karlsruhe Institute of Technology, Karlsruhe, Germany
4 Institute of Applied Physics (APH) and Center for Functional Nanostructures (CFN), Karlsruhe Institute of Technology, Karlsruhe, Germany
5 Department of Physics, University of Illinois at Urbana-Champaign, Urbana, Il, USA
∗ E-mail: johannes.stegmaier@kit.edu

1 Installation of Prerequisites

The first step to use the fast C++ implementation of the segmentation algorithm (v0.1) is to download CMake from `http://www.cmake.org/`. This tool is used to build the Insight Toolkit (ITK) libraries, which are freely available for download under `http://www.itk.org/` and later for building the segmentation executable itself. Additionally, the Qt libraries are needed, which can be obtained from `http://qt-project.org/`. The following Qt modules are essential: QtCore, QtGui, QtXML and QtXMLPatterns. Detailed installation instructions for ITK and Qt are given on the respective webpages. For development and testing of the software, we used CMake 2.8, ITK v4.3 and Qt v4.8.2 under Scientific Linux 5 using GCC 4.1.2. However, it should also be straightforward to use it with upcoming releases of these software packages. The software has also been successfully compiled under Windows 7 using Microsoft Visual Studio 2012 and its associated C++ compiler.
HINTS:

- In case you want to be able to process `tiff`-files larger than 4GB it is necessary to have a BigTiff compatible `libtiff` version installed as well as enabling the ITK CMake flag `ITK_USE_64BIT_IDS` during the ITK makefile generation (See `http://bigtiff.org/`).
- It is crucial to compile both ITK and Qt libraries and executables in 64 bit if large images should be used.
- We recommend to store ITK in at most two subfolders to the system root, otherwise path limits from the file system may be exceeded.

2 Compiling the Segmentation Implementation

After having installed and compiled the prerequisites, it should be possible to compile the segmentation implementation. This can again be achieved using the CMake build-tool and your favored compiler using the `CMakeLists.txt` located in the folder `ProjectRoot/projects/RealToxMiner/projects/CMake/`. Therefore, the source path of CMake has to be set to `ProjectRoot/projects/RealToxMiner/projects/CMake` and the build path has to be set e.g. to `ProjectRoot/projects/RealToxMiner/projects/CMake/Build` (See Fig. 1, folder names are denoted relative to the installation directory). If ITK and Qt are not found automatically, make sure to redirect CMake to the correct paths where you stored the respective libraries.

After successful Makefile generation using CMake it should be possible to compile the segmentation algorithm, e.g., using the `make` command within a Linux terminal or to use the generated Visual Studio project files.

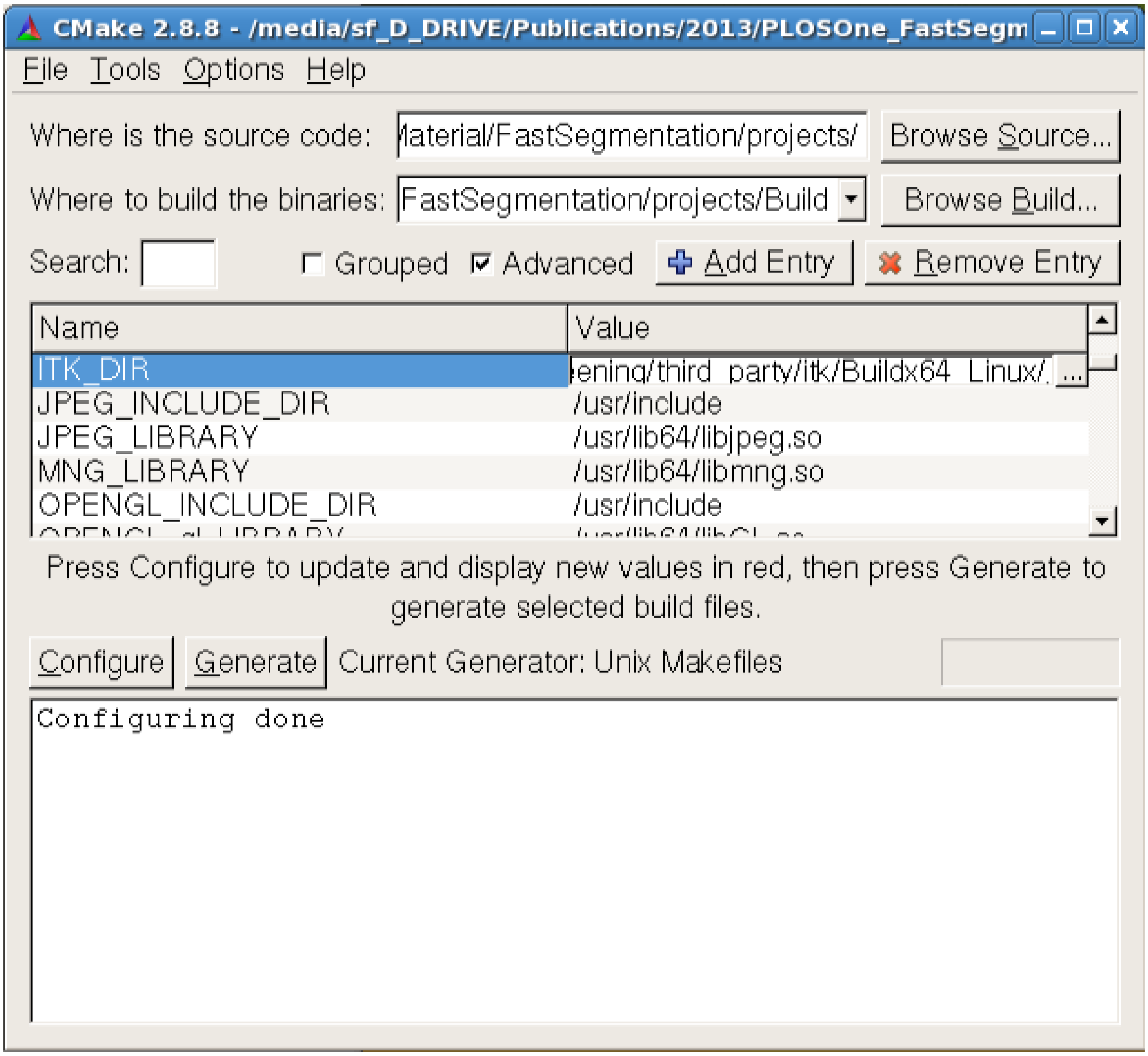

Figure 1. CMake settings to build the segmentation implementation on Linux.

3 Application Example

Once the executable has successfully been compiled, it can be started via the Linux terminal application with the following call: `./RealToxMiner < input.txt` or in the Windows command prompt using `RealToxMiner < input.txt`, where `input.txt` is a single-line text file that determines the input and output parameters for the executable. Note that the executable itself is called without parameters but all inputs are piped either with a file or directly. The application assumes the following input parameters in the input text file, separated by a single space:

- `OutPath`: The folder used to write result images and log files to.
- `InFileName`: Absolute path for the input filename.
- `OutFileName`: Filename of the generated output file(s).
- `XMLFile`: Absolute path to the XML pipeline that should be used for processing.
- `RandomSeed`: Random integer (will used to prevent simultaneous starting of the processing on clusters). Disabled in the provided implementation.

In the `examples` folder you find three small 3D datasets that show labeled nuclei of a zebrafish embryo. Input files for the compiled executable as well as the XML pipeline file for the segmentation are also given there. Be sure to adjust the absolute paths within the input files according to the specific location on your disk. If everything went fine, the specified output folder should contain a log file including the processing times, parameters and pipeline components as well as the generated result images like shown in Fig. 2. All parameters can be adjusted in the file `ProjectRoot/examples/data/twangsegmentation.xml` using a simple text editor. A description of the valid parameters is given as comments in this XML file. The execution of the segmentation performs a validity check of the provided XML file and creates an associated XSD file. Note, that the executable tries to start processing even if the XML is not valid. Errors of to the XML validation according to ID's can be ignored, as they are not yet relevant.

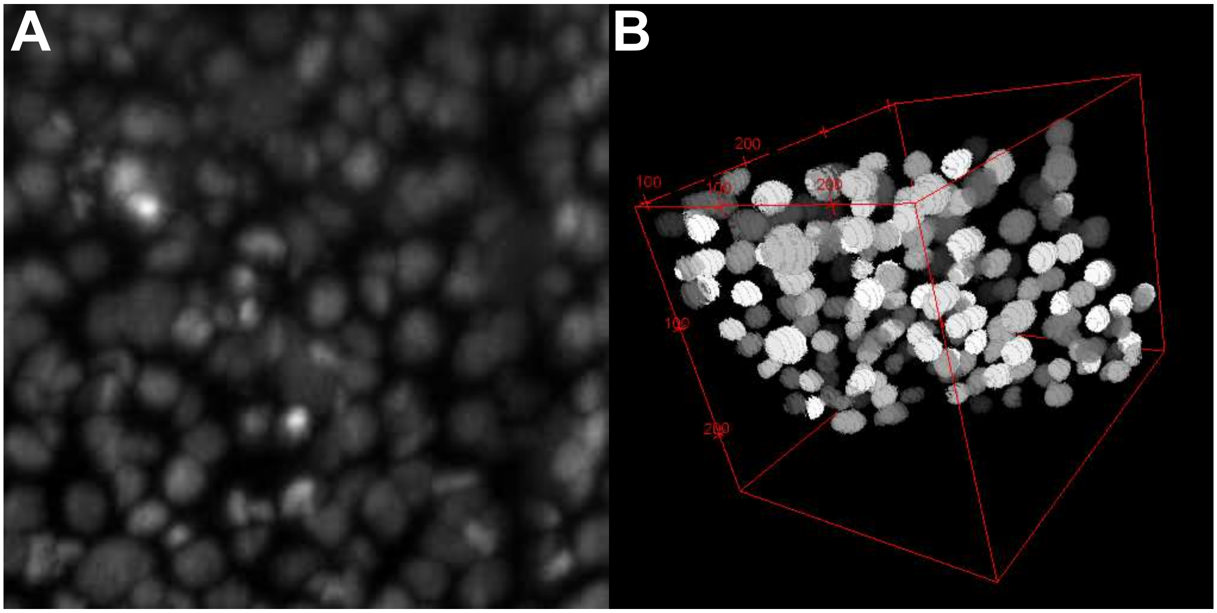

Figure 2. Segmentation results using the input file `ProjectRoot/examples/danio_test2.txt` that internally uses the image file `ProjectRoot/examples/data/danio_test2.tif` and the XML processing pipeline `ProjectRoot/examples/data/twangsegmentation.xml`. The maximum projection is shown in (A) and the segmentation result is shown in (B).
