# Supplementary figures and images for: Fast Segmentation of Stained Nuclei in Terabyte-Scale, Time Resolved 3D Microscopy Image Stacks
Source: PLoS One. 2014 Feb 27;9(2):e90036. doi: 10.1371/journal.pone.0090036 (PMC3937404; doi:10.1371/journal.pone.0090036)

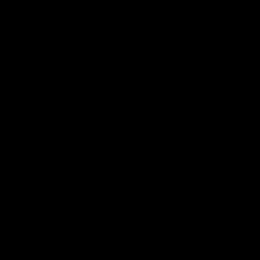

Supplement: File S1 — Implementation of the TWANG Segmentation Algorithm. C++ source code of the fast segmentation pipeline presented in this paper. The provided archive contains all sources, installation instructions and an example image. (ZIP) [file pone.0090036.s001.zip › Software_S1/examples/data/danio_test2.tif]

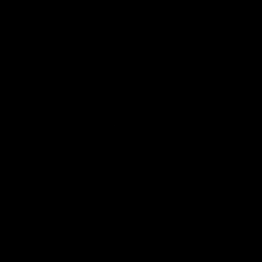

Supplement: File S2 — Example Data of a Zebrafish Embryo. The archive contains two additional cropped regions of a 3D DSLM image of a developing zebrafish embryo. (ZIP) [file pone.0090036.s002.zip › ExampleData_S1/examples/data/danio_test1.tif]

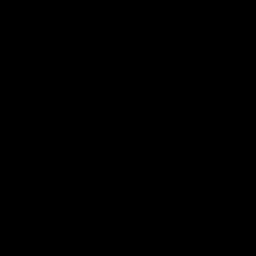

Supplement: File S2 — Example Data of a Zebrafish Embryo. The archive contains two additional cropped regions of a 3D DSLM image of a developing zebrafish embryo. (ZIP) [file pone.0090036.s002.zip › ExampleData_S1/examples/data/danio_test3.tif]
